# Evolutionary Biology for the 21st Century

**DOI:** 10.1371/journal.pbio.1001466

**Published:** 2013-01-08

**Authors:** Jonathan B. Losos, Stevan J. Arnold, Gill Bejerano, E. D. Brodie, David Hibbett, Hopi E. Hoekstra, David P. Mindell, Antónia Monteiro, Craig Moritz, H. Allen Orr, Dmitri A. Petrov, Susanne S. Renner, Robert E. Ricklefs, Pamela S. Soltis, Thomas L. Turner

**Affiliations:** 1 Museum of Comparative Zoology and Department of Organismic and Evolutionary Biology, Harvard University, Cambridge, Massachusetts, United States of America; 2 Department of Zoology, Oregon State University, Corvallis, Oregon, United States of America; 3 Departments of Developmental Biology and Computer Science, Stanford University, Stanford, California, United States of America; 4 Department of Biology, University of Virginia, Charlottesville, Virginia, United States of America; 5 Department of Biology, Clark University, Worcester, Massachusetts, United States of America; 6 Department of Molecular and Cellular Biology, Harvard University, Cambridge, Massachusetts, United States of America; 7 Department of Biochemistry and Biophysics, University of California, San Francisco, California, United States of America; 8 Department of Ecology and Evolutionary Biology, Yale University, New Haven, Connecticut, United States of America; 9 Museum of Vertebrate Zoology, University of California, Berkeley, California, United States of America; 10 The Australian National University, Canberra, Australia; 11 Department of Biology, University of Rochester, Rochester, New York, United States of America; 12 Department of Biology, Stanford University, Stanford, California, United States of America; 13 Department of Biology, University of Munich, Munich, Germany; 14 Department of Biology, University of Missouri, St. Louis, Missouri, United States of America; 15 Florida Museum of Natural History, University of Florida, Gainesville, Florida, United States of America; 16 Department of Ecology, Evolution and Marine Biology, University of California, Santa Barbara, California, United States of America

## Abstract

New theoretical and conceptual frameworks are required for evolutionary biology to capitalize on the wealth of data now becoming available from the study of genomes, phenotypes, and organisms - including humans - in their natural environments.

## Introduction

We live in an exciting time for biology. Technological advances have made data collection easier and cheaper than we could ever have imagined just 10 years ago. We can now synthesize and analyze large data sets containing genomes, transcriptomes, proteomes, and multivariate phenotypes. At the same time, society's need for the results of biological research has never been greater. Solutions to many of the world's most pressing problems—feeding a global population, coping with climate change, preserving ecosystems and biodiversity, curing and preventing genetically based diseases—will rely heavily on biologists, collaborating across disciplines.

Theodosius Dobzhansky famously proclaimed that “nothing makes sense in biology except in the light of evolution." Though Dobzhansky's statement is sometimes dismissed by biologists in other fields as self-promotion, recent advances in many areas of biology have shown it to be prophetic. For example, genomics, which emerged mostly from molecular biology, is now steeped in evolutionary biology. Evolutionary theory helps to explain our origins, our history, and how we function as organisms and interact with other life forms, all of which are crucial to understanding our future (e.g., [1]–[5]). Evolutionary approaches have helped reconstruct the history of human culture, including, for example, the history of human populations and languages [6]–[11]. And the impact of evolutionary biology is extending further and further into biomedical research and nonbiological fields such as engineering, computer sciences, and even the criminal justice system.

The pervasive relevance of evolution can be seen in the 2009 report commissioned by the National Research Council of the National Academies, *A New Biology for the 21^st^ Century* [12], which identified four broad challenges for biology: develop better crops to feed the world, understand and sustain ecosystem function and biodiversity in a changing world, expand sustainable alternative energy sources, and understand individual health. In each of these areas, the report noted, evolutionary concepts and analyses have played—and will continue to play—an integral role.

It's hard to overstate evolutionary biology's power to explain the living world and our place in it. Many applications of evolutionary theory and methods—from animal and plant breeding to vaccine development to management of biological reserves and endangered species—affect society and promote human well-being [13],[14]. Much human activity, however, is changing Earth's climate and habitats, with uncertain but potentially severe environmental stresses on many other species [15]–[18], and the solutions to the many resulting problems may well require understanding evolutionary interactions among species and their mutual dependencies.

**Figure 1:**
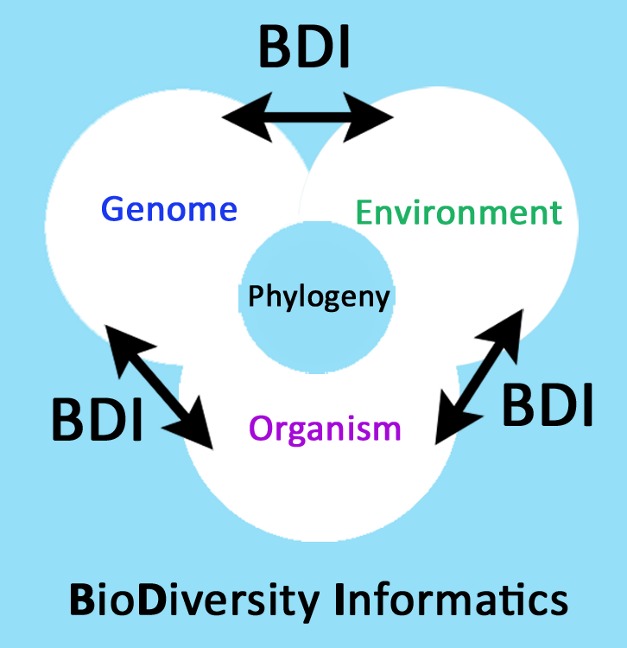
Evolutionary biology is being transformed by increasing access to burgeoning data on variation in genomes, organisms, and the environment. All this can be connected to the Tree of Life (phylogeny), from populations to entire clades, and is enabled by new protocols and networks in biodiversity informatics.

Our ability to apply evolutionary concepts to a wide range of problems has never been greater. Changes in the availability of data and an emerging scientific culture that embraces rapid, open access to many kinds of data (genomic, phenotypic, and environmental), along with a computational infrastructure that can connect these rich sources of data ([19], Figure 1), will transform the nature and scale of problems that can be addressed by evolutionary biology.

Periodically, groups of scientists meet to identify new opportunities in evolutionary biology and associated disciplines (e.g., [12],[20]–[23]). Rather than set a specific research agenda for the future—clearly the charge of individual investigators—the aim has been to identify new themes and research directions that are already emerging in the field and to focus on the intersection of fundamental problems with new technologies and methods. In the following sections, we briefly highlight some key applications of evolutionary biology, provide examples of emerging research areas, and identify infrastructure and training needs.

## Evolutionary Applications

### Evolutionary Medicine

The new field of “evolutionary medicine" [24]–[26] posits that understanding our evolutionary past can inform us of the causes of perplexing common diseases. For instance, diabetes and autoimmune diseases such as asthma may represent mismatches between evolutionary adaptation to the environments in which humans evolved and current conditions. In addition, some age-related conditions, such as cancer, can be understood as the outcome of selection for early reproduction, when humans faced dying of disease or predation at an early age. This long-term selection on the cellular machinery to optimize growth and survival through early reproduction may now explain the prevalence of cancer late in life, a modern malaise that emerges because of the recent, rapid extension of postreproductive lifespan [27]. Aside from providing explanations for the occurrence of diseases, the field of evolutionary medicine is also concerned with suggesting strategies for slowing the evolution of resistance in pathogen populations [28]–[30]; strategies to improve public health and reduce the incidence of common diseases [31],[32]; prediction of diseases that may emerge from recent host-shifts to humans [33]; discovery, design, and enhancement of drugs and vaccines (e.g., [34]); and understanding the role of the microbiome in human health [35].

### Feeding the Human Population

Feeding the rapidly growing human population, especially with increasing stress on agricultural systems from climate change, continues to be a major challenge. The green revolution, from the 1950s onwards, rested on selective plant breeding for larger yields and was underpinned by evolutionary theory [36]. Currently, the trend is to rely on biotechnology to introduce either herbicide resistance genes or herbivore-directed toxins, such as Bt, to combat crop competitors and herbivores, respectively, and thus promote increasing yields [37]. Unfortunately, genetically modified crops are genetically uniform and so do not represent a long-term solution against the evolution of either herbicide or Bt resistance. In addition, these herbicide resistance or toxin genes can be transferred to other nontarget species through pollen-mediated hybridization, rendering them resistant or toxic as well [38]. The agriculture of the future must incorporate evolutionary thinking to reduce the evolution of resistance and the risk of pathogen outbreaks. Maintaining genetic diversity in crop and animal production systems considerably reduces these risks [38].

### Sustaining Biological Diversity

Evolutionary approaches have often been applied to the conservation of species and ecosystems [13],[39]–[42]. Linking spatial data on phenotypes, genomes and environments in a phylogenetic context allows us to identify and name Earth's diverse life forms. This linkage, in turn, helps to provide the basic units needed to quantify taxonomic diversity and to pursue its conservation. Determining phylogenetic relationships among species allows us to identify their unique adaptations and provides the historical context to understand how they arose [43]–[45]. Evolutionary approaches also can be used to determine the origins of invasive species [46]–[48] and to help design effective remediation [49],[50]. Collectively, understanding the distribution of current biodiversity and its evolutionary response to past environmental change is fundamental to mitigating effects of ongoing habitat loss and climate change [51]. Given the rate of anthropogenic climate change, evolutionary theory and experiments can help predict vulnerability (i.e., inability to adapt) of species and thus improve conservation strategies [52].

### Computation and Design

Models of mutation, inheritance, and selection have inspired the development of computational evolutionary algorithms that are used to solve complex problems in many fields [53],[54]. In particular, engineering and design processes have incorporated evolutionary computation, leading to improvements in design of cars, bridges, traffic systems robots, and wind turbine energy, among other applications [55]–[59].

### Evolution and Justice

Genealogical relationships bear on many court cases. Is the defendant really the parent of the plaintiff? Does the evidence (e.g., blood, semen, or skin cells) at the crime scene tend to exonerate or implicate a suspect? Evolutionary methods, particularly population genetics, are now used frequently in forensics and court cases to test the link of crime scene evidence to individuals [60], and phylogenetic analyses have been vetted and accepted as valid and appropriate methods for determining facts of history in the United States criminal court system [61].

## Emerging Research and Future Challenges in Evolutionary Biology

Divining the direction of future scientific research is always fraught with difficulty. Nonetheless, we feel that it is possible to identify some general themes that will be important in coming years. We also present some examples of classic research problems that remain unsolved and that might well be the focus of future work, as well as new and important questions for which evolutionary approaches may be key.

### New Theory

The flood of data in all areas of evolutionary biology poses important theoretical challenges: new kinds of theory are sometimes required to make sense of new kinds of data. We can already point to certain broad areas of evolutionary biology that will likely demand sustained theoretical work. These include the elaboration of more formal theories for evolutionary developmental biology (e.g., analysis of gene network evolution and modification); the more complete incorporation of the roles of epigenetics, behavior, and plasticity in models of trait evolution; analysis of units of selection; and attempts to construct a quantitative and predictive theory that describes the genetic basis of adaptation. In other areas, problems will likely be more statistical than theoretical. Indeed, the enormous quantity of genome data poses serious statistical challenges even for fields that already possess strong theoretical foundations, such as evolutionary genetics.

### The Explosion and Diversity of Data

DNA sequencing can now generate whole-genome data not only for single representatives of a few species but for multiple individuals from multiple conspecific populations and even from entire communities. Such multilevel data are giving rise to whole new fields of study (e.g., population genomics and metagenomics) as well as to new theoretical, computational, and data management challenges.

One particularly exciting avenue of research afforded by new genomic technology is the possibility of directly observing the dynamics of evolution. In the last few years, genomic analyses of experimental evolution have yielded new understanding of how RNA molecules, viruses, and bacteria evolve (bacteria: [62],[63]; virus: [64]; RNA molecules: [65]). This approach is now being applied to eukaryotic model systems such as *C. elegans* and yeast [66]–[68]. These efforts will continue to expand and will surely involve natural systems in field settings. Past evolution, for example, can be inferred from samples derived from ancient specimens, archived material in museum collections, lake sediments, and glacier cores. Contemporary evolution can be inferred from genomic sampling across seasons and years and can be detected in response to climatic disturbances such as El Niño events and to manmade environmental changes such as oil spills. In parallel with long-term ecological data (e.g., species abundance and distributions through time), we can now track genomic variation through ecological and evolutionary time. This capability, together with the realization that evolutionary change can occur on ecological timescales [69], provides an important new window on real-time evolution. Evolution on contemporary time scales is likely to be especially important in the context of evolving pathogens, pest resistance, and the response of organisms to rapid environmental change.

**Figure 2:**
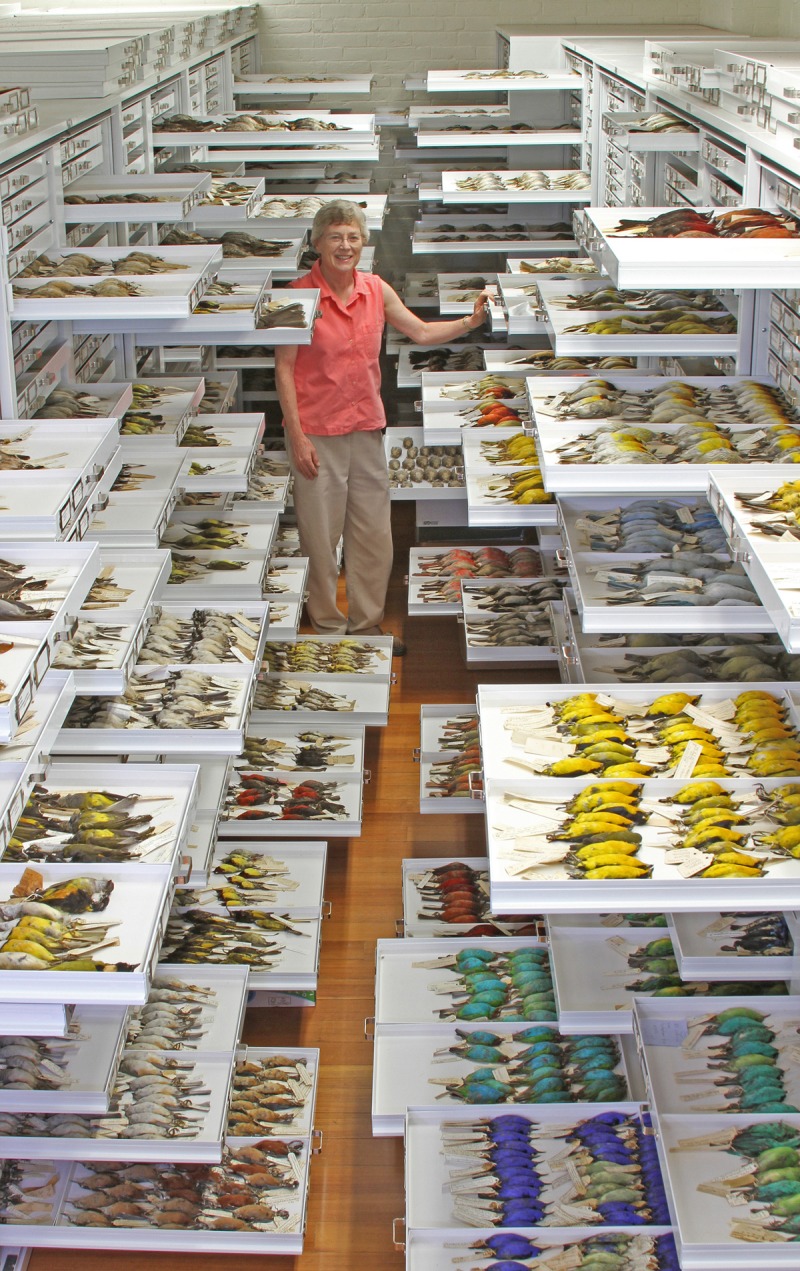
Natural history museum collections are tremendous repositories of specimens and data of many sorts, including phenotypes, tissue samples, vocal recordings, geographic distributions, parasites, and diet. Photo by Jeremiah Trimble, Department of Ornithology, Museum of Comparative Zoology, Harvard University.

While the explosion of data on genome sequences has received the most attention, supplementing these data with information on the natural history of individuals, species, and their environments will be important. Core information from field-collected specimens always includes species identity and place and time of collection, but increasingly, this information is being enriched with links to field notes and phenotypic (e.g., images), behavioral (e.g., sounds), and genomic data in a variety of databases (e.g., Morphbank—http://www.morphbank.net/, Barcode of Life—http://www.barcodeoflife.org/, Macaulay Library—http://macaulaylibrary.org/). Precise information on place, time, and reproductive stage can be integrated with data on local environmental conditions, often obtained from remote sensing [70]. The key is to connect information across repositories, such as natural history museums and genomic databases (Figure 2). Such efforts will also include observational data provided by the broader public [71].

### Evolutionary Processes That Shape Genomic and Phenotypic Variation

The availability of genomic data from a remarkable range of species has allowed the alignment and comparison of whole genomes. These comparative approaches have been used to characterize the relative importance of fundamental evolutionary processes that cause genomic evolution and to identify particular regions of the genome that have experienced recent positive selection, recurrent adaptive evolution, or extreme sequence conservation [72]–[75]. Yet more recently, resequencing of additional individuals or populations is also allowing genome-wide population genetic analyses within species [76]–[82]. Such population-level comparisons will allow even more powerful study of the relative importance of particular evolutionary processes in molecular evolution as well as the identification of candidate genomic regions that are responsible for key evolutionary changes (e.g., sticklebacks [83], butterflies [84], *Arabidopsis* [85]). These data, combined with theoretical advances, should provide insight into long-standing questions such as the prevalence of balancing selection, the relative frequency of strong versus weak directional selection, the role of hybridization, and the importance of genetic drift. A key challenge will be to move beyond documenting the action of natural selection on the genome to understanding the importance of particular selective agents. For example, what proportion of selection on genomes results from adaptation to the abiotic environment, coevolution of species, sexual selection, or genetic conflict? Finally, as sequencing costs continue to drop and analytical tools improve, these same approaches may be applied to organisms that present intriguing evolutionary questions but were not tractable methodologically just a few years ago. The nonmodel systems of today may well become the model systems of tomorrow [86].

### Earth–Biosphere Interactions Over Vast Stretches of Time and Space

We are in the midst of a massive perturbation of natural communities as species respond to human-driven changes in climate and land cover. Beyond the challenge of understanding the capacity of species to respond (e.g., [51],[87]) and the potential for dramatic state-shifts in the biosphere [17] lies the daunting problem of understanding the many interactions between community-scale ecological dynamics and evolution of traits within populations.

We now can also ask if evolution matters for ecosystem functioning. To date, most ecosystem studies have assumed that all individuals that compose a population within a community are equivalent ecologically. But individuals within a population are variable, and this variation may lead to ecological interactions that are in a continual state of evolutionary flux as ecologically driven evolutionary change feedbacks to alter the ongoing ecological interactions [88]–[90]. This evolutionary perspective on communities is an emerging area that will require the acquisition and analysis of large, temporal samples of genomic and phenotypic data, as well as the direct measurement of fitness. Samples that include paleo/historical DNA as well as contemporary DNA might be especially valuable by providing a temporal view on such questions.

### Understanding Biological Diversification

A major and urgent challenge is to improve knowledge of the identity and distribution of species globally. While we need to retain the traditional focus on phenotypes, powerful new capabilities to obtain and interpret both genomic and spatial data can and should revolutionize the science of biodiversity. Building on momentum from single-locus “barcoding" efforts, new genome-level data can build bridges from population biology to systematics [91]. By establishing a comprehensive and robust “Tree of Life," we will improve understanding of both the distribution of diversity and the nature and timing of the evolutionary processes that have shaped it.

Studies of the biodiversity of Bacteria and Archaea are complicated by the widespread occurrence of lateral gene transfer. However, the phylogeny of these organisms and their genes remains critical to understanding their scope, origins, distributions, and change over time [92]. The advent of metagenomic sequencing of environmental microbial communities has revealed greater diversity and flux of genotypes than ever imagined, defying conventional species concepts and presenting a major challenge to applying traditional evolutionary and ecological theory to understanding microbial diversity [93],[94]. Addressing this challenge will be necessary to advance microbial ecology beyond the descriptive stage. Moreover, it is only with such understanding that a natural history of microbes can be developed, leading to more meaningful exploration of genomic structure and function, the origin of novel genes, and increased knowledge of microbial influences at both the global and individual (microbiome) levels.

In addition to documenting biodiversity, more research is needed on the processes that produce this diversity. While research on speciation has seen a resurgence over the last two decades [95]–[97], new tools—including genomic data—can support new approaches for a number of important questions, including discovering genomic signatures underlying the evolution of prezygotic reproductive isolation, and describing how hybridization, contact between incipient species, genome reorganization, and genome duplication, affect speciation.

Understanding the diversification of species and the origin of adaptations poses a number of challenges for evolutionary biologists, including integration of the fossil record with diversification inferred from the relationships among contemporary species; determining the relationship between lineage and phenotypic diversification; understanding the factors that lead to the replacement of clades over time; understanding the occupancy of ecological niche space through evolutionary diversification, adaptive radiation, and extinction; and assessing the role that evolving species interactions play in diversification.

**Figure 3:**
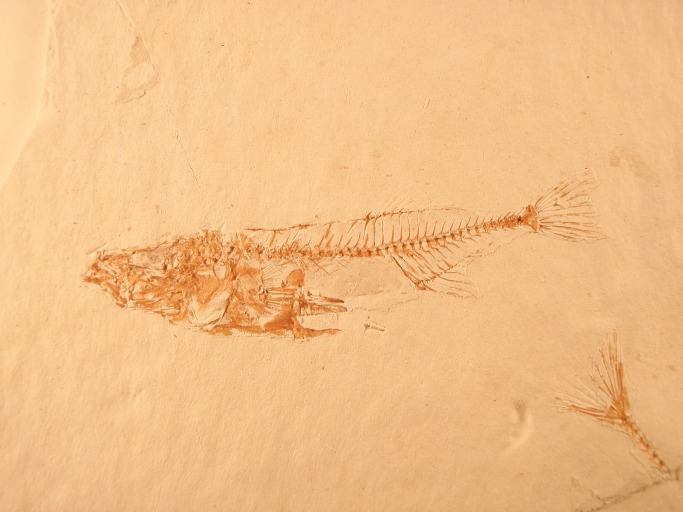
Developing genetic and evolutionary tools for taxa with an extensive fossil record will be an important means of integrating the study of evolutionary pattern and process. Genomic sequence data for stickleback fish is now providing insight into evolutionary patterns, such as the reduction in the pelvic skeleton, manifest both in the fossil record and in extant populations [83]. Photograph courtesy Peter J. Park.

All evolution has an ecological context that is essential to the interpretation of diversification. Consequently, we need to incorporate analyses of the environmental context of evolution, particularly species interactions that are likely to both set limits to diversification and promote evolutionary novelty. For all these reasons, further integration of paleontology with other fields of evolutionary biology, as well as development of genetic-evolutionary research programs on clades with excellent fossil records (e.g., foraminifera, diatoms, mollusks; Figure 3), will be important. More generally, uniting understanding of evolutionary pattern and process will require reductionist studies on evolutionary mechanisms of species formation and phenotypic change, as well as broadly historical studies that incorporate phylogenetic, paleontological, and geological data.

As we address these challenges, the importance of natural history data cannot be overemphasized. Observations on the natural history of organisms, the basic building blocks of more detailed studies of ecology and evolution, are critical if we are to preserve and understand biological diversity [98]. Though few would argue against this point in principle, natural history research is rarely encouraged or supported. Making the acquisition of natural history data an integral part of hypothesis-driven science is now more important than ever.

## Logistical Issues and Opportunities

To take full advantage of technological advances, especially the availability of new data types and databases, we must confront several challenges that involve community resources and how we use them. Some challenges concern infrastructure, while others involve aspects of scientific culture. Still others involve how we train the next generation of evolutionary biologists, who will need a better grasp of diverse disciplines—from natural history to developmental biology—as well as bioinformatics skills to handle immense datasets across multiple fields (see Text S1 and also Figure S2).

The infrastructure challenges center on creation of new kinds of databases—for instance, ones that focus on (continuous) phenotypic and not merely (discrete) DNA sequence data—as well as on integration across databases to allow synthesis of very different kinds of data (see Text S2). The cultural challenges center on the need for supporting a climate of scientific openness. Maintaining openness will require evolutionary biologists to make the results of their research available rapidly and in a form that is most useful to their colleagues. The scientific community has already made great strides in this direction (for instance by requiring deposition of data as a condition for publication and by founding open access journals), but additional steps are necessary. We strongly support the movement toward open access for the scientific literature to accelerate research and allow more investigators to participate. We also encourage provision of open software, data and databases, as well as their computational reuse and distillation, as outlined by Lathrop et al. (2011) [99]. These individual and community efforts will be increasingly necessary for development of new research programs and insights.

As noted at the outset, we live in an exciting time for evolutionary biology. The field has embraced the “omic" revolution, and answers to many classic questions, which have motivated research for a century, are now within reach. The study of evolution, which in the past was often equated with changes in gene frequencies in populations, has become more holistic and integrative. Researchers are increasingly interested in exploring how interactions among genes, individuals, and environments have shaped the evolutionary process, both at micro- and macrolevels. At the same time, large challenges such as global warming, novel infectious diseases, and threats to biodiversity are increasing, and the opportunity for evolutionary biologists to contribute to their resolution has never been greater.

Realizing the full potential inherent in evolutionary biology is, however, far from assured. The task of integrating evolutionary knowledge within and across scales of biological organization, as discussed above, requires development of many comparative databases and analytical tools. We would do well to collaborate broadly, cultivating new expertise, and to watch out for the unexpected, as analyses of new kinds of data can reveal that preconceptions are unfounded.

Because most of our science is supported by limited public funds, evolutionary biologists and ecologists should support and participate in efforts to help the public understand the issues and the value of scientific understanding. Science in general and evolutionary science in particular are often politicized, exactly because of their fundamental importance to human society. The next 20 years hold the promise of a golden age for evolutionary biology. Whether we realize that promise depends in part on how effectively we communicate that message.

**Glossary**

**Cyberinfrastructure**—The research environments that support advanced data acquisition, data storage, data management, data integration, data mining, data visualization, and other computing and information processing services distributed over the Internet beyond the scope of a single institution. In scientific usage, cyberinfrastructure is a technological solution to the problem of efficiently connecting laboratories, data, computers, and people.

**Evolutionary developmental biology**—The study of the evolution of development, often by the comparative study of gene expression patterns through the course of development in different species.

**Evolutionary genetics**—Population and quantitative genetics.

**Gene network**—A flow diagram describing the interactions among genes during development that affect a particular phenotype or set of phenotypes.

**Genomics**—The study of the entire complement of DNA in organisms (Genome), including is sequence and organization.

**GMO**—Genetically modified organisms in which the genome has been deliberately changed; transgenic organisms resulting from DNA manipulations.

**Lateral (horizontal) gene transfer**—Genetic transfer between species, as opposed to vertical gene transmission from parents to offspring in a lineage.

**Metadata**—Data associated with individual DNA sequences or organismal specimens (e.g., the date and locality where the sample originated, its ecological context, etc.).

**Model organism**—Organisms whose genome has been sequenced and for which sophisticated tools for genetic manipulation are available.

**Natural history**—The entire description of an organism, including its phenotype, genome, and ecological context (i.e., abiotic niche as well as its biotic interactions with other species).

**Nonmodel organism**—Organisms whose genome has not been sequenced and/or for which sophisticated tools for genetic manipulation are not available.

**Ontology**—The naming of categories, especially of the functions of genes.

**Population genetics**—The study of the evolutionary forces that change the genetic composition of a population; the discipline is often concerned with evolution at one or a few genetic loci.

**Quantitative genetics**—The study of the inheritance and evolution of traits that are typically affected by many genetic loci.

**Transgenic tools**—Tools that enable the deliberate transfer of DNA sequences from one organism to another or the deletion or modification of DNA sequences, in every cell, in one organism.

## Supporting Information

Figure S1 An example of the enormous phylogenetic trees that soon will represent the norm in phylogenetic analyses. This is the consensus tree of the maximum likelihood phylogenies for 55,473 species of seed plants with the location of significant shifts in species diversification rates marked in red across the tree. Adapted from [4].
(TIF)

Figure S2 The Phenomobile, a remote sensing field buggy, and the Blimp, for remotely imaging an entire field. The Phenomobile integrates a variety of remote sensing technologies for measuring phenotypic variables on many plants simultaneously. The buggy straddles a plot and collects measurements of plant temperature, stress, chemistry, color, size and shape, as well as measures of senescence. The Blimp is designed to image all the plants in an entire field from a height of 30–80 m using both infrared and digital color cameras. These technologies were developed by David Deery of the High Resolution Plant Phenomics Centre at the Commonwealth Scientific and Industrial Research Organisation in Australia. Photo credit: Carl Davies, CSIRO Plant Industry.
(TIF)

Text S1 Training to sustain evolutionary biology.
(DOCX)

Text S2 Infrastructure needs and opportunities in evolutionary biology.
(DOCX)
