# Supplementary figures and images for: Evolutionary Biology for the 21st Century
Source: PLoS Biol. 2013 Jan 8;11(1):e1001466. doi: 10.1371/journal.pbio.1001466 (PMC3539946; doi:10.1371/journal.pbio.1001466)

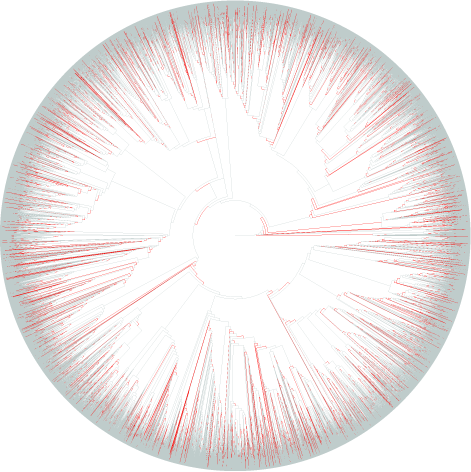

Supplement: Figure S1 — An example of the enormous phylogenetic trees that soon will represent the norm in phylogenetic analyses. This is the consensus tree of the maximum likelihood phylogenies for 55,473 species of seed plants with the location of significant shifts in species diversification rates marked in red across the tree. Adapted from [4]. (TIF) [file pbio.1001466.s001.tif]

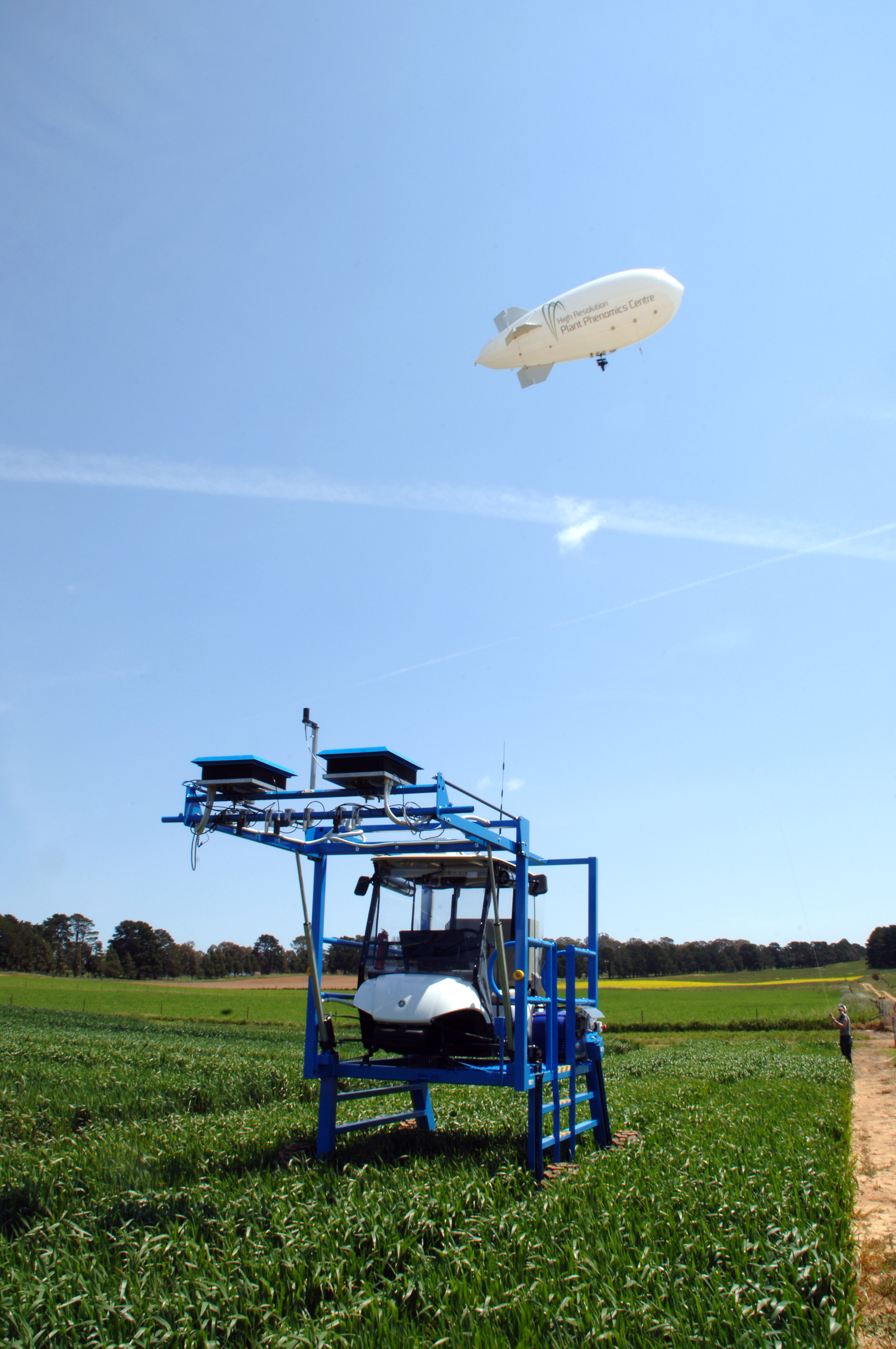

Supplement: Figure S2 — The Phenomobile, a remote sensing field buggy, and the Blimp, for remotely imaging an entire field. The Phenomobile integrates a variety of remote sensing technologies for measuring phenotypic variables on many plants simultaneously. The buggy straddles a plot and collects measurements of plant temperature, stress, chemistry, color, size and shape, as well as measures of senescence. The Blimp is designed to image all the plants in an entire field from a height of 30–80 m using both infrared and digital color cameras. These technologies were developed by David Deery of the High Resolution Plant Phenomics Centre at the Commonwealth Scientific and Industrial Research Organisation in Australia. Photo credit: Carl Davies, CSIRO Plant Industry. (TIF) [file pbio.1001466.s002.tif]
