# Supplementary material for: Evolutionary Biology for the 21st Century
Source: PLoS Biol. 2013 Jan 8;11(1):e1001466. doi: 10.1371/journal.pbio.1001466 (PMC3539946; doi:10.1371/journal.pbio.1001466)
Supplement: Text S1 — Training to sustain evolutionary biology. (DOCX) [file pbio.1001466.s003.docx]

**S1. Training to sustain evolutionary biology**

Contemporary evolutionary biology integrates fields as diverse as genomics, informatics, systematics, evolutionary genetics, paleontology, and developmental biology. However, few individuals receive rigorous training in all these areas as part of their education, and many practicing scientists are struggling to retool as new technologies emerge. In addition to an “informatics divide,” which prevents organismal biologists from making full use of the flood of genomic information, we also face a “natural history divide,” which sometimes prevents genomicists and informaticians from identifying and addressing the most biologically relevant questions. Similarly, insufficient quantitative skills and awareness of relevant theory prevents many organismal biologists from using appropriate tools to address their questions. Accordingly, a pressing need exists for interdisciplinary training and networking for evolutionary biologists at all career stages. It is unrealistic to expect students to become experts across all domains. Nevertheless, students need deep expertise in one or more areas, and sufficient knowledge of others to understand new developments and to collaborate effectively with relevant experts. Below, we describe four examples of relevant training activities for students, educators, and practicing scientists.

(1) Training opportunities for graduate students and post-docs—including independent post-doctoral fellowships that are not tied to a Principal Investigator – that emphasize interdisciplinary training. Many students, for example, need training in the basics of computer programming and mathematical modeling.

(2) Opportunities to provide training in new technologies for scientists working in isolated settings, such as curators in museums and teachers at small colleges. Such individuals are at risk of losing contact with cutting-edge developments in evolutionary biology, which could limit their research productivity and reduce the impact of their teaching.

(3) Workshops to promote the integration of organismal biology and natural history with genomics, genetics, and bioinformatics. Evolutionary and organismal biologists—and the professional societies that represent them—should reach out to the genomics and bioinformatics communities to find and exploit opportunities to share information and provide cross-training.

(4) Development of undergraduate curricula that integrate evolution, bioinformatics and genomics and all of these with biomedical curricula. Exposure to applications of evolutionary biology, such as those in medicine and climate-change science, training in data management, and opportunities for research experiences at biological field stations should also be offered in undergraduate programs. More generally, we emphasize the need to train liberally educated citizens who have an awareness and appreciation of the diverse disciplines that affect, and that are affected by, evolutionary biology.
