# Supplementary material for: Evolutionary Biology for the 21st Century
Source: PLoS Biol. 2013 Jan 8;11(1):e1001466. doi: 10.1371/journal.pbio.1001466 (PMC3539946; doi:10.1371/journal.pbio.1001466)
Supplement: Text S2 — Infrastructure needs and opportunities in evolutionary biology. (DOCX) [file pbio.1001466.s004.docx]

**S.2 Infrastructure Needs and Opportunities in Evolutionary Biology**

Technological advances, particularly in genomics and bioinformatics, now allow us to approach fundamental evolutionary questions in new ways and, in some cases, to answer them definitively. Just as importantly, some advances, especially the availability of new data types and databases, open the door to entirely new questions about evolutionary processes.

To take full advantage of these technological advances, we must confront several challenges that involve community resources and how we use them. Some of these challenges concern infrastructure, while others involve aspects of scientific culture. The infrastructure challenges center on creation of new kinds of databases—for instance, ones that focus on (continuous) phenotypic and not merely (discrete) DNA sequence data, as well as on integration across databases. Evolutionary biologists must be able to work with and synthesize very different kinds of data.

The cultural challenges center on the need for supporting a climate of scientific openness. Maintaining openness will require evolutionary biologists to make the results of their research available rapidly and in a form that is most useful to their colleagues. The scientific community has already made great strides in this direction (for instance by requiring deposition of data as a condition for publication and by founding open access journals), but additional steps are necessary. We strongly support the movement toward open access for the scientific literature to accelerate research and allow more investigators to participate. We also encourage provision of open software, data and databases, as well as their computational reuse and distillation, as outlined by Lathrop et al. (2011) [1]. These individual and community efforts will be increasingly necessary for development of new research programs and insights.

**Cyberinfrastructure**

Solving synthetic questions in evolutionary biology will require investment in developing and implementing cyberinfrastructure that will unite disparate and complex data sources in user-friendly ways. The broad scope of biological data—genomic, transcriptomic, phenotypic, image-based, ecological—means that we must revise current software and analytical tools and develop new methods to integrate, navigate, and analyze large, diverse data sets. These needs are precipitated by the explosion of sequence data and made even more acute with the addition of biological metadata.

The need for effective visualization tools pervades all data-intensive areas of evolutionary biology. For example, current software cannot effectively display the Tree of Life or comparative genome structure on a large scale. Software for estimating phylogeny can now handle thousands of leaves (e.g., species, individuals, sequences), but appropriate tools for displaying the results or for mapping traits over large phylogenies have not been developed adequately (Figure S1). Likewise, mapping the current and predicted geographic distribution of a species is certainly possible, but scaling to all species in a community or across a phylogeny remains cumbersome. Integrated visualization—for example of phylogenies of genes or lineages in relation to geography and through time ([2], [3])—holds great potential. Finally, tools for visualizing and comparing genomes may accommodate a small set of similar genomes, but do not adequately handle scaling to more divergent genome structures from many species.

Evolutionary biology is a synthetic science, with data drawn from disparate sources. As complex databases are constructed, it is tempting to conceptualize one that houses all relevant data for a given problem. However, a more tractable solution is that of independent but integrated databases. For example, many of the estimated one billion specimens housed in U.S. natural history collections are tied to additional data and resources, such as morphological or behavioral measurements, genetic data, ecological information, images, and video and audio recordings. Development of data standards and distributed data systems, along with a sociological transformation towards open data sharing, are making biodiversity data available to the scientific community and the public (see [5], [6] and references therein). Much of the data in museum collections can serve as a foundation for phenotypic, distributional, and ecological data sets that can be linked with genetic and genomic data in integrated evolutionary studies.

Of course, a cyber-network of biodiversity information can only be as strong and as rich as the collections from which biological data ultimately flow. It is therefore critical that efforts to develop a cyberinfrastructure for evolutionary biology be linked to support for biodiversity surveys and infrastructure for museums, herbaria and collections of living materials, such as culture collections and botanical gardens.

**Infrastructure for Large-Scale Phenotypic Analysis**

It is increasingly clear that we need to mount an extensive effort to collect and organize phenotypic data ([7]). In comparison to our growing database of genomes, our knowledge of phenotypes remains inadequate. This discrepancy results in part from the fact that phenotype space has many more dimensions than genotype space ([8]). However, our success in linking genotype and phenotype is dependent on our ability to measure phenotypes both accurately and consistently, as well as the development of ontologies that relate cell types, developmental programs, morphologies, behaviors, and other traits across individuals and species (e.g., see phenoscape.org, an effort to expand ontologies from the zebrafish community to teleosts as a whole). High-throughput image acquisition and analysis and associated morphometrics, all with accompanying databases (e.g., [9], [10]), enable the linking of macroevolution-level collections of fossils, microevolution-level data collected from natural populations, and experimental-level altered morphologies of mutants. Commercial facilities now specialize in large-scale phenotypic analysis and are well equipped to do high-throughput screens for rare mutants (e.g., greenhouses that can raise thousands of seedlings) or, alternatively, multi-phenotype screens for a mutant strain (e.g., hundreds of behavioral assays run on a few mutant/transgenic animals). Such means are revolutionizing the scale at which geneticists design and perform experiments (e.g., [11]; Figure S2), although the development of statistical methods capable of dealing with such high dimensional data still remains a challenge. Evolutionary biologists are just now beginning to capitalize on these approaches (e.g., [12]), but it is easy to imagine how they can be adapted to address questions about evolutionary diversification and constraint.

As cyberinfrastructure expands, the need to sustain these resources increases. How will databases and related cyberinfrastructure be maintained in perpetuity? Who will host and maintain databases and cyberinfrastructure, and what is the appropriate funding model? Attention to long-term sustainability must accompany the development of new cyberinfrastructure to ensure resources for maintenance, curation, and continued use of data.

1. Lathrop RH, Rost B, ISCB Membership, ISCB Executive Committee, ISCB Board of Directors et al. 2011. ISCB public policy statement on open access to scientific and technical research literature. PLoS Comput Biol 7: e1002014. doi: 10.1371/ journal.pcbi.1002014.
2. Guralnick R, Hill A (2009) Biodiversity informatics: automated approaches for documenting global biodiversity patterns and processes. Bioinformatics 25: 421-428. doi:10.1093/bioinformatics/btn659
3. Page RDM (2012) Space, time, form: viewing the Tree of Life. Trends Ecol Evol 27: 113-120.
4. Smith S, Beaulieu J (2011) Understanding angiosperm diversification using small and large phylogenetic trees. Am J Bot 98: 404-414.
5. Constable H, Guralnick R, Wieczorek J, Spencer C, Peterson AT (2010) VertNet: a new model for biodiversity data sharing. PLoS Biol 8: e1000309. doi: 10.1371/ journal.pbio.1000309
6. Mindell DP, Fisher BL, Roopnarine P, Eisen J, Mace GM, et al. (2011) Aggregating, tagging and integrating biodiversity research. PLoS ONE 6: e19491. doi:10.1371/journal.pone.0019491.
7. Hoekstra HE (2010) Evolutionary Biology: the next 150 years. In: Bell MA, Futuyma DA, Eanes WF, Levinton JS, editors. Evolution since darwin: the first 150 years. Sunderland: Sinauer Press.
8. Houle D (2010)  Numbering the hairs on our heads: The shared challenge and promise of phenomics. PNAS 107:1793-1799. doi:10.1073/pnas.0906195106.
9. Ayroles JF, Carbone MA, Stone EA, Jordan KW, Lyman RF, et al. (2009) Systems genetics of complex traits in *Drosophila melanogaster*. Nat Genet 41: 299–307. doi:10.1038/ng.332.
10. Harbison ST, Carbone MA, Ayroles JF, Stone EA, Lyman RF, et al. (2009) Co-regulated transcriptional networks contribute to natural genetic variation in Drosophila sleep. Nat Genet 41: 371–375. doi:10.1038/ng.330.
11. Tecott, LH, Nestler TJ (2004) Neurobehavioral assessment in the information age. Nature Neuro 7: 462-466. doi:10.1038/nn1225.
12. Zhang X, Hauss RJ, Borevitz JO. Natural genetic variation for growth and development revealed by high-throughput phenotyping in *Arabidopsis* thaliana. G3 2:29-34. doi:10.1534/g3.111.001487.

Figure Legends

**Figure S1**. An example of the enormous phylogenetic trees that soon will represent the norm in phylogenetic analyses. This is the consensus tree of the maximum likelihood phylogenies for 55,473 species of seed plants with the location of significant shifts in species diversification rates marked in red across the tree. Adapted from [4].

**Figure S2**. The Phenomobile, a remote sensing field buggy, and the Blimp, for remotely imaging an entire field. The Phenomobile integrates a variety of remote sensing technologies for measuring phenotypic variables on many plants simultaneously. The buggy straddles a plot and collects measurements of plant temperature, stress, chemistry, color, size and shape, as well as measures of senescence. The blimp is designed to image all the plants in an entire field from a height of 30-80 m using both infrared and digital color cameras. These technologies were developed by David Deery of the High Resolution Plant Phenomics Centre at the Commonwealth Scientific and Industrial Research Organisation in Australia. Photo credit: Carl Davies, CSIRO Plant Industry.
